# Acute ocular toxicity study of *Chandrodaya Varti Anjana* in albino rabbits: A safety assessment

**DOI:** 10.1016/j.jaim.2026.101363

**Published:** 2026-07-08

**Authors:** Sangita Shekhaliya, Narayan Bavalatti, Manjusha Rajagopala

**Affiliations:** Department of Shalakya Tantra, All India Institute of Ayurveda, New Delhi, India

**Keywords:** Acute ocular toxicity, Albino rabbits, *Chandrodaya varti anjana*, Herbo-mineral

## Abstract

Cataract is a clouding of the lens, that normally affects vision. It is the leading cause of reversible blindness and visual impairment globally. *Chandrodaya Varti Anjana*, a classical Ayurvedic herbo mineral formulation, is widely used in ophthalmic practice for conditions such as *Timira* (∼blurred vision), *Arma* (∼pterygium) and *Kacha* (∼opacity of lens). Despite its clinical acceptance, toxicological evaluation of its ocular safety is limited. Aim of the study is to evaluate the acute ocular toxicity of *Chandrodaya Varti Anjana* in albino rabbits. Albino rabbits were included for the study after approval of IAEC. As per the OECD guidelines 0.1 g of testing drug was placed in the conjunctival sac of one eye each animal. The lids were then gently held together for 2 s. Throughout the 21-days study period, none of the test animals exhibited conjunctival corneal, or iridial reactions and no signs of chemosis were observed. Acute ocular toxicity study indicates that topical application of *Chandrodaya Varti Anjana* was well-tolerated in albino rabbits without any signs of ocular toxicity. These findings provide a preliminary safety profile that supports the potential for future clinical evaluation in humans. However, further studies with larger animal samples and controlled human clinical trials are necessary to establish definitive safety and efficacy.

## 1 Introduction

Cataract is an opacification of the lens, that impairs vision [1]. Globally, cataracts are the leading cause of blindness and the second-leading cause of moderate to severe visual impairment. Over 100 million people worldwide are affected by cataracts, with 17 million cases resulting in blindness [2]. Cataract surgery is often expensive, requiring specialized equipment and trained medical professionals. A preventive or reversal drug could treat cataracts in their early stages, halting progression before vision is severely impaired. Therefore, pharmacological treatment for cataracts is a cheaper and more readily available option for patients, which is also a hot topic for years. Some studies suggest that delaying the onset of cataracts by just 10 years could halve the need for surgery [3].

In *Ayurveda*, variety of *Anjana* (∼collyrium) formulations described for *Timira* or *Kacha* (∼opacity of lens). Among them, *Chandrodaya Varti Anjana* is frequently prescribed for conditions such as *Timira* (∼blurred vision), *Mamsavriddhi* (∼Pterygium), *Kacha* (∼opacity of lens) and night blindness [4]. *Chandrodaya Varti* is a herbomineral preparation described in *Bhaisajya Ratnavali, Netraroga Adhikar* contains *Maricha* (∼*Piper nigrum* Linn.), *Pippali*(∼*Piper longum* Linn.), *Haritaki*(∼*Terminalia chebula* Ritz.), *Vacha*(∼*Acorus calamus* Linn), *Kustha*(∼*Saussurea lappa* C.B. Clarke), *Bibhitaki*(∼*Terminalia bellirica* Roxb.), *Shankhanabhi*(∼*Convolvulus pluricaulis* Chois.), *Manahshila*(∼Red Arsenic - As2S2) and *Ajadugdha*(∼goat milk).

Although *Chandrodaya Varti Anjana* is widely used in Ayurvedic ophthalmic practice and its therapeutic indications are well documented in classical texts, there is paucity of systematic toxicological data evaluating its ocular safety profile. Most available literature focuses on its clinical efficacy; however, standardized preclinical safety assessments in accordance with internationally accepted regulatory guidelines are lacking. This represents a significant evidence gap, particularly because the formulation contains herbo-mineral ingredients such as *Manahshila*, which may raise concerns regarding potential local irritation or toxicity. Furthermore, ocular tissues are highly sensitive and direct topical exposure may produce immediate irritant or corrosive reactions. Therefore, systematic scientific documentation and in vivo testing are essential to complement traditional knowledge, ensuring that these formulations meet modern evidence-based safety criteria for wider clinical acceptance. In vitro assays and theoretical safety assumptions are insufficient to fully simulate the complex physiological responses of the intact eye, including corneal transparency, conjunctival vascular reactions and iridial involvement. Therefore, it was planned to conduct an in vivo study to see whether ‘*Chandrodaya Varti Anjana*’ causes any acute eye irritation/corrosion effect in albino rabbits, which are widely accepted models for ophthalmic safety evaluation. Such evaluation is essential not only to bridge the translational gap between traditional claims and modern safety standards but also to ensure evidence-based validation of its ophthalmic use. This toxicity study was approved by the IAEC. Therefore, in compliance with OECD guidelines for testing of chemicals, section 4, Test no. 405: Acute Eye Irritation/Corrosion test guidelines, an acute eye irritation/corrosion test study with rabbit was conducted. [5]. The results were interpreted based on Harmonised Integrated Classification System for Human Health and Environmental Hazards of Chemical Substances and Mixtures (14 August, 2001) [6].

## 2 Objectives

To evaluate the safety profile of *Chandrodaya Varti Anajna* in albino rabbits.

## 3 Methodology

Ethical approval for the acute eye irritation/corrosion test in rabbits was taken from the Institutional Animal Ethical Committee (IAEC Approval letter number: NIA/IAEC/2024/90) before initiating the test. The study was conducted under the strict compliances of animal house and husbandry conditions of animal house, at National Institute of Ayurveda, Jaipur. The details of the protocol are as follow:

### 3.1 Study centre

Animal House, National Institute of Ayurveda, Jaipur.

### 3.2 Test formulation

Chandrodaya Varti Anjana.

### 3.3 Source of formulation

Procured from GMP Certified Pharmaceuticals (Indian Medicines Pharmaceutical Corporation Limited, a Unit of Government of India Enterprise)

**Formulation type:** *Gutika Anjana*.

**Batch no.:** AVA 003.

**Manufacture Date:** 08/2024.

**Date of Exp.:** 07/2027.

### 3.4 Animal

Albino Rabbits.

### 3.5 Number of animals

03.

### 3.6 Experimental guidelines

OECD 405 guidelines for ocular toxicity/irritation was followed.

### 3.7 Animal husbandary condition

During study period animal was kept under controlled environmental conditions. The room temperature kept between 19.1 °C and 21.6 °C, with humidity maintained between 40% and 58%, and a 12-h light–dark cycle. Air was exchanged 10-15 times per hour. Animals were individually housed in clean stainless-steel cages (approximately 450 × 600 × 450 mm), which were regularly cleaned as per in-house procedures. They were fed UV-irradiated standard commercial pelleted rabbit feed and supplied RO-treated water in polycarbonate bottles with stainless-steel nozzles. Only healthy animals were selected after examination, and acclimatization was carried out for 8–12 days prior to test item instillation.

### 3.8 Procedures

A total 3 female rabbits, 10-11 weeks old of body weight 1.2-1.8 kg were selected. A veterinary health check was performed. Selected healthy animals was acclimatized for 8 days for initial test and 12 days for confirmatory test respectively prior to test item instillation. On day 1 of acclimatization, each animal was identified by permanent marking (inside ear pinna) using marker pen and by cage card labeling. The initial test was performed on a one animal and confirmatory test was performed on two animals. The eyes of the rabbits were examined using a standard light source and ophthalmoscope within 24 h prior to treatment to rule out pre-existing abnormalities such as pain, congestion, redness, infection, dryness or excessive secretion and found normal. Approximately 60 min (±5 min) prior to test item instillation, 0.01 mg/kg buprenorphine was administered by subcutaneous injections and 5 ± 1 min prior to test item installation, two drop of a topical ocular anesthetics 0.5% proparacaine hydrochloride was administrated in each eye. 8 h ± 30 min post-test item instillation, 0.01 mg/kg buprenorphine and 0.5 mg/kg meloxicam were administered subcutaneously to provide a continued therapeutic level of systemic analgesia [7].

The pH of the test item was measured before test item application, and it was found to be 4.6 which was within acceptable range [8]. The test item, *Chandrodaya Varti Anjana*, was prepared by trituration using a sterile mortar and pestle to obtain a fine, uniform powder, as per OECD 405 recommendations to prevent mechanical irritation. A standardized dose of 0.1 g was utilized as recommended by the guideline for solid substances to ensure adequate conjunctival exposure. Drug was instilled into the conjunctival sac of the right eye after gently retracting the lower eyelid, while the left eye served as an untreated control. To reduce substance loss, the eyelids were gently held closed for about 2 s.

The test item was administered as a single-dose application on day 0, and no repeated dosing was performed, as per OECD 405 guidelines for acute ocular toxicity study. The study followed a sequential testing strategy. An Initial Test was first performed on a single animal to screen for severe reactivity. Since no corrosive or severe irritant effects were observed within the observation period for the first animal, a Confirmatory Test was subsequently conducted on two additional rabbits to validate the findings. This sequential approach ensures that the minimum number of animals is exposed to a potentially painful substance, adhering to the 3Rs (Replacement, Reduction and Refinement) principle of animal welfare. The response was graded after 30 min, 1 h, 2 h, 4 h, 8 h, 24 h, 48 h, 72 h, day 7, day 14 and day 21. Test animals were routinely evaluated for the entire duration of the study for clinical signs of pain and/or distress (e.g. repeated pawing or rubbing of the eye, excessive blinking, excessive tearing) [9] [10].

## 4 Observation

The grades of ocular reaction (conjunctivae, iris, cornea, chemosis) were observed and recorded as per the grading of ocular lesion mentioned in OECD guideline 405,2012.

### 4.1 Initial test (Animal no. 1)

Conjunctivae of grade 0 “normal” was observed after 30 min, 1 h, 2 h, 4 h, 8 h, 24 h, 48 h, 72 h, day 7, day 14 and day 21. Cornea of grade 0 “No ulceration or opacity” was observed after 30 min, 1 h, 2 h, 4 h, 8 h, 24 h, 48 h, 72 h, day 7, day 14 and day 21. Iris of grade 0 “normal” was observed after 30 min, 1 h, 2 h, 4 h, 8 h, 24 h, 48 h, 72 h, day 7, day 14 and day 21. Chemosis of grade 0 “normal” was observed after 30 min, 1 h, 2 h, 4 h, 8 h, 24 h, 48 h, 72 h, day 7, day 14 and day 21.

No severe ocular lesions were observed in initial test, hence, confirmatory test was performed with two additional animals.

### 4.2 Confirmatory test (Animal no. 2 and 3)

In both animals, conjunctivae found normal (grade 0) at 30 min, 1 h, 2 h, 4 h, 8 h, 24 h, 48 h, 72 h, day 7, day 14 and day 21. Iris of grade 0 “normal” was observed at 30 min, 1 h, 2 h, 4 h, 8 h, 24 h, 48 h, 72 h, day 7, day 14 and day 21. Cornea of grade 0 “No ulceration or opacity” was observed at 30 min, 1 h, 2 h, 4 h, 8 h, 24 h, 48 h, 72 h, day 7, day 14 and day 21. Chemosis found “normal (grade 0)” in both the animals at 30 min, 1 h, 2 h, 4 h, 8 h, 24 h, 48 h, 72 h, day 7, day 14 and day 21.

The untreated eyes (left eye) of all the three rabbits were normal throughout the observation period.

There were no clinical signs of systemic toxicity such as no tremors, no altered behavior, no convulsion, no weight loss, no respiratory distress, no diarrhea and no mortality was observed in any of the treated animals.

## 5 Results

**Fig. 1:**
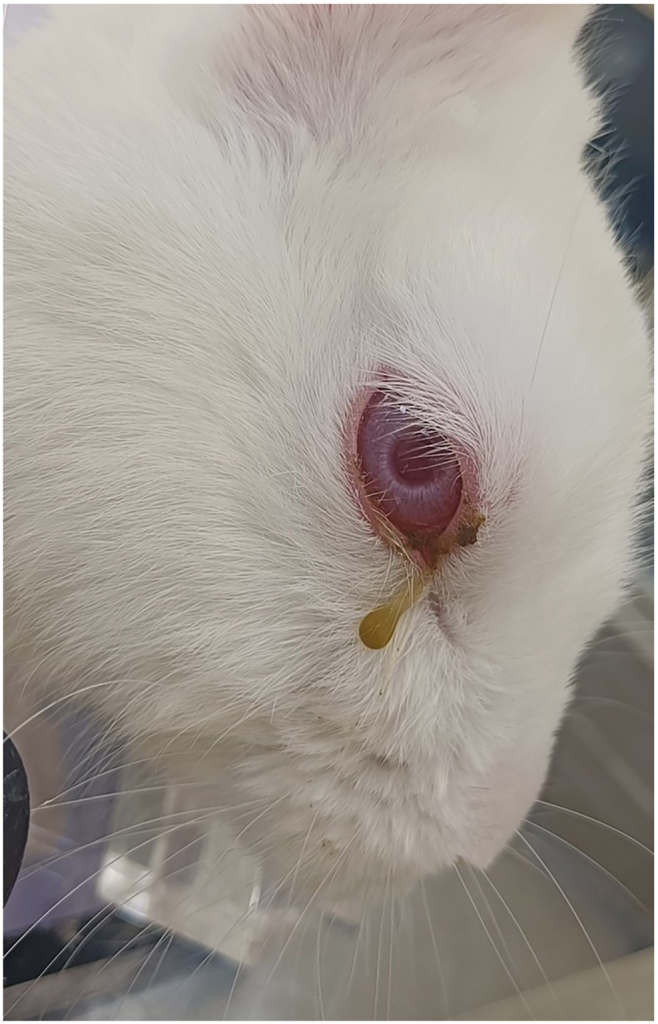
Observation After 30 min of instillation CVA.

**Fig. 2:**
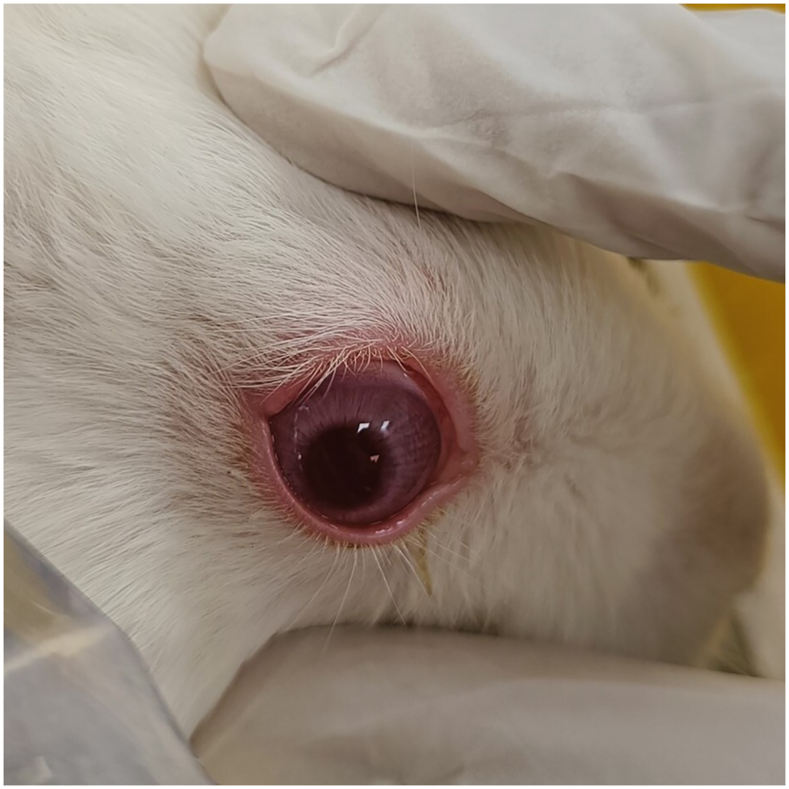
Observation After 1 h of instillation CVA.

**Fig. 3:**
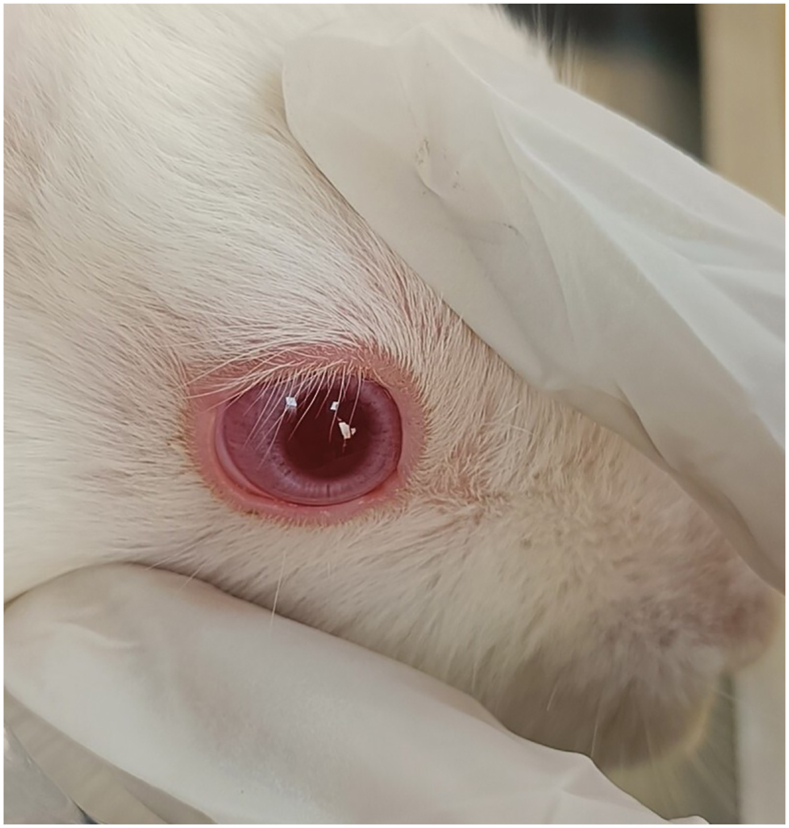
Observation After 24 h of instillation CVA.

**Fig. 4:**
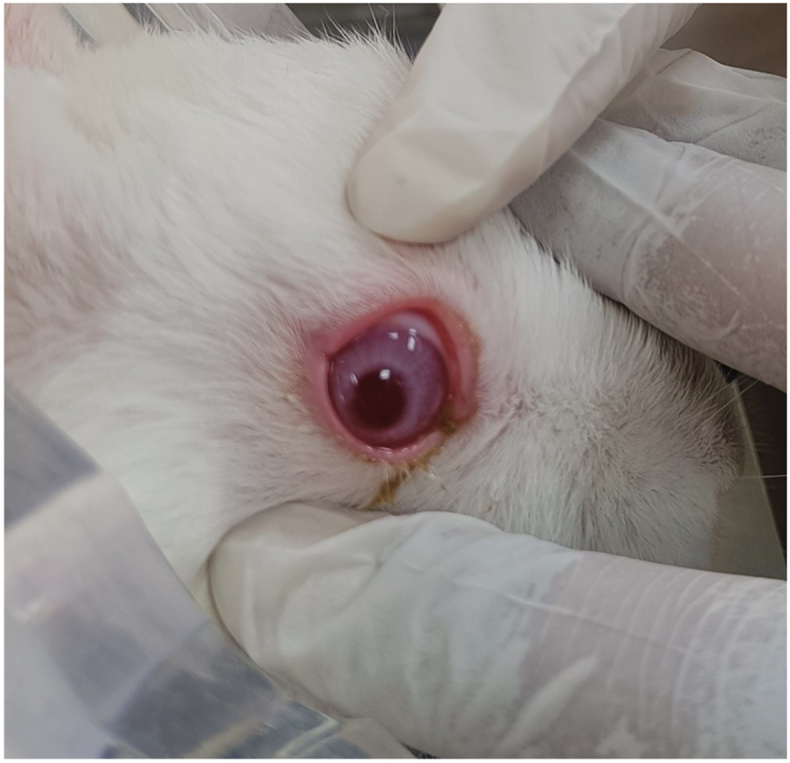
Observation After 7 days of instillation CVA.

**Fig. 5:**
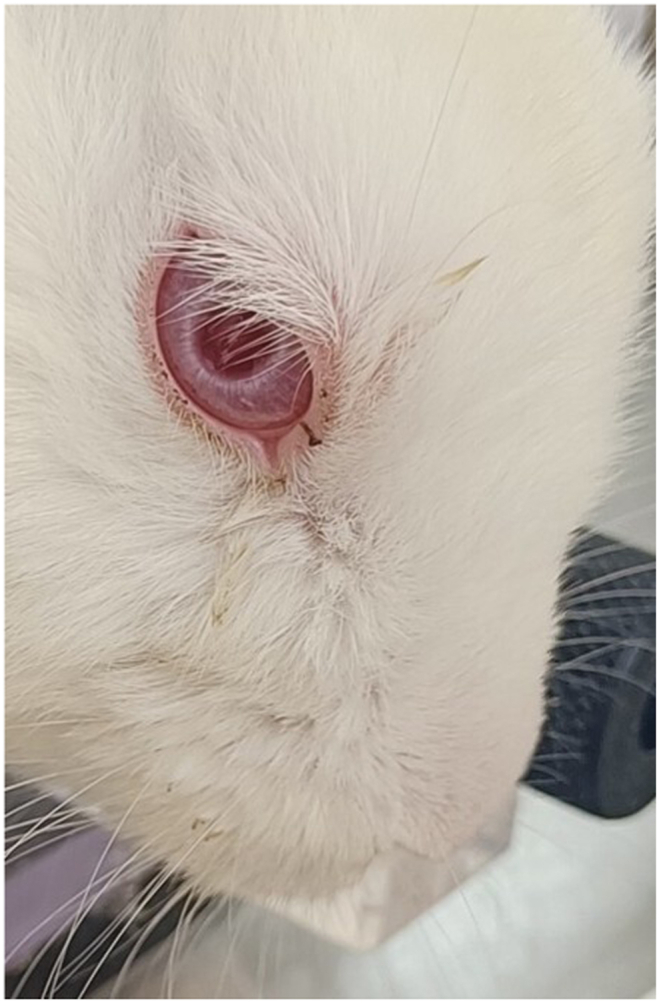
Observation After 14 days of instillation CVA.

**Fig. 6:**
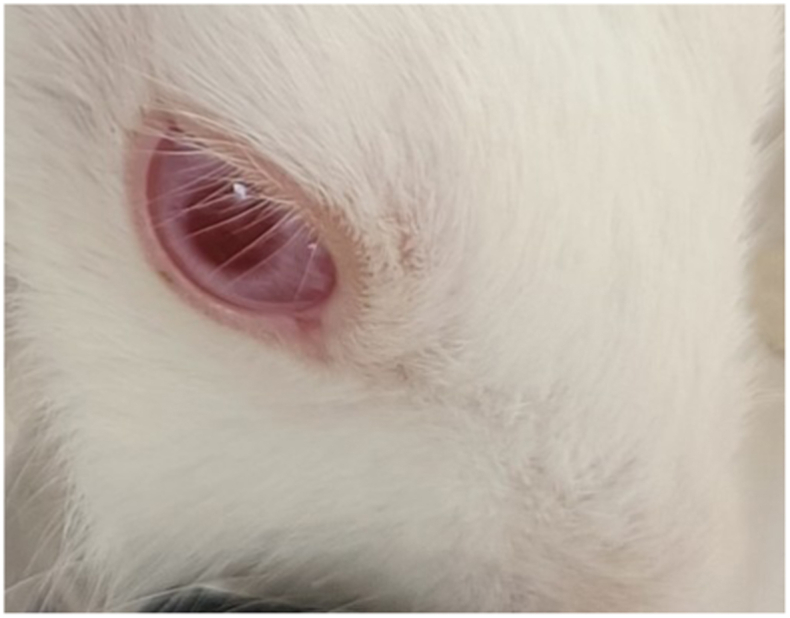
Observation After 21 days of instillation CVA.

Throughout the 21-days study period, none of the test animals exhibited corneal, iridial or conjunctival reactions, and no signs of chemosis were observed (Table 1) (Fig. 1, Fig. 2, Fig. 3, Fig. 4, Fig. 5, Fig. 6)

**Table 1:** Individual scores for ocular irritation.

Parameter	Animal no	Pre treatment	Time of observation							
			30 min	1 h	24 h	48 h	72 h	7 days	14 days	21 days
**Cornea**	1	0	0	0	0	0	0	0	0	0
	2	0	0	0	0	0	0	0	0	0
	3	0	0	0	0	0	0	0	0	0
**Iris**	1	0	0	0	0	0	0	0	0	0
	2	0	0	0	0	0	0	0	0	0
	3	0	0	0	0	0	0	0	0	0
**Conjunctivae**	1	0	0	0	0	0	0	0	0	0
	2	0	0	0	0	0	0	0	0	0
	3	0	0	0	0	0	0	0	0	0
**Chemosis**	1	0	0	0	0	0	0	0	0	0
	2	0	0	0	0	0	0	0	0	0
	3	0	0	0	0	0	0	0	0	0

## 6 Discussion

*Chandrodaya Varti* includes ingredients with *Tikta*(∼bitter), *Kashaya*(∼Astringent), *Katu*(∼pungent) *Rasa, Laghu, Ruksha, Tikshna Guna* and *Lekhaniya* properties, making it suitable for treating eye disorders like Timira or *Kanch, Mamsavriddhi*. However, the inclusion of ingredients such as *Manahshila* and *Shankhnabhi* necessitates a critical discussion regarding its acute ocular toxicity. As the *Manahshila*, which contains the heavy metal arsenic, which is the most hazardous substance among the top 20 listed by the Agency for Toxic Substances and Disease Registry (ATSDR) [11]. *Shankhnabhi* may cause eye irritation, pain, redness and inflammation, but acute ocular toxicity study on the formulation is not readily available. For that reason, acute ocular toxicity study of *Chandrodaya Varti* was essential.

Based on the observations recorded according to OECD Test Guideline 405, *Chandrodaya Varti Anjana* was classified as “non-irritant” in albino rabbits. The findings indicate that the formulation did not produce acute ocular irritation under the experimental conditions of the study. However, as the present investigation was limited to acute ocular toxicity assessment in an animal model, further studies including sub-acute, chronic toxicity evaluation, and well-designed clinical trials are necessary to establish its long-term safety and therapeutic applicability in human subjects.

## 7 Conclusion

Based on above results, ocular application of *Chandrodaya Varti Anjana* in New Zealand White Rabbit did not produce any acute ocular irritant effects under the tested conditions. Hence, the test item of *Chandrodaya Varti Anjana* maybe considered “Non-irritant.” However, this finding requires further validation through studies with a larger sample size.

## Authors contribution

SS: Conceptualization, writing, review and editing. MR and NB: Conceptualization, guidance, supervision, review and validation. All authors have read and agreed to the published version of the manuscript.

## Declaration of generative AI in scientific writing

Artificial intelligence (AI) was not used for this research work.

## Sources of funding

There was no financial support used for this research work.

## Conflictof interest

None.
